# The Performance of Equations That Estimate Glomerular Filtration Rate against Measured Urinary Creatinine Clearance in Critically Ill Patients

**DOI:** 10.1155/2021/5520653

**Published:** 2021-05-18

**Authors:** Hasan M. Al-Dorzi, Abdulmajeed A. Alsadhan, Ayman S. Almozaini, Ali M Alamri, Hani Tamim, Musharraf Sadat, Lolowa Al-Swaidan, Elwaleed Elhassan, Yaseen M. Arabi

**Affiliations:** ^1^College of Medicine, King Saud Bin Abdulaziz University for Health Sciences, King Abdullah International Medical Research Center, Intensive Care Department, King Abdulaziz Medical City, Ministry of National Guard-Health Affairs, Riyadh, Saudi Arabia; ^2^College of Medicine, King Saud Bin Abdulaziz University for Health Sciences, King Abdullah International Medical Research Center, Internal Medicine Department, King Abdulaziz Medical City, Ministry of National Guard-Health Affairs, Ministry of National Guard-Health Affairs, Riyadh, Saudi Arabia; ^3^Department of Internal Medicine, American University of Beirut, Beirut, Lebanon; ^4^College of Pharmacy, King Saud bin Abdulaziz University for Health Sciences, King Abdullah International Medical Research Center, Pharmaceutical Care Department, King Abdulaziz Medical City, Ministry of National Guard-Health Affairs, Riyadh, Saudi Arabia

## Abstract

The performance of glomerular filtration rate- (GFR-) estimating equations was studied against creatinine clearance measured by 24-hour urine collection (CrCl_24h-urine_) in critically ill patients. *Methods*. In this substudy of the PermiT trial (https://clinicaltrials.gov/ct2/show/ISRCTN68144998), patients from King Abdulaziz Medical City-Riyadh who had CrCl_24h-urine_ were included. We estimated GFR using Cockroft–Gault (CG), modification of diet in renal disease study (MDRD), chronic kidney disease epidemiology collaboration (CKD-EPI), and Jelliffe equations. For the CG equation, we entered the actual weight in one calculation (CG_actual-wt_), and if BMI ≥30 kg/m^2^, we entered the ideal body weight (CG_ideal-wt_) and the adjusted body weight (CG_adjusted-wt_) in two calculations. We calculated the MDRD equation based on 4 (MDRD-4) and 6 variables (MDRD-6). The performance of these equations was assessed by different ways including Spearman correlation, bias (difference between estimated GFR and CrCl_24h-urine_), precision (standard deviation of bias), and Bland–Altman plot analysis. *Results*. The cohort consisted of 237 patients (age 45 ± 20 years, males 75%, mechanically ventilated 99% with serum creatinine 101 ± 94 *µ*mol/L and CrCl_24h-urine_ 108 ± 69 ml/min/1.73 m^2^). The correlations between the different equations and CrCl_24h-urine_ were modest (*r*: 0.62 to 0.79; *p* < 0.0001). Bias was statistically significant for CG_actual-wt_ (21 ml/min), CG_adjusted-wt_ (12 ml/min), and MDRD-6 (-10 ml/min) equations. Precision ranged from 46 to 54 ml/min. The sensitivity of equations to correctly classify CrCl_24h-urine_ 30–59.9 ml/min/1.73 m^2^ was 17.2% for CG_actual-wt_, 30.0% for CG_ideal-wt_, 31.0% for CG_adjusted-wt_, 31.0% for MDRD-4, 39.1% for MDRD-6, 13.8% for CKD-EPI, and 34.5% for Jelliffe equation. *Conclusions*. Commonly used GFR-estimating equations had limited ability to properly estimate CrCl_24h-urine_ and to correctly classify GFR into clinically relevant ranges that usually determine dosing of medications.

## 1. Introduction

Appropriate dosing of medications is frequently dependent on renal function. The Kidney Disease Improving Global Outcomes (KDIGO) clinical practice guidelines consider GFR as the preferred measure of kidney function rather than serum creatinine (Cr) and recommend estimating GFR in most circumstances and measuring it when greater accuracy is required [1]. To accurately measure GFR, exogenous substances, such as inulin, are used as filtration markers [2]. Despite being the gold standard for assessment of renal function, this measurement is not routinely performed in clinical practice as it is complex, impractical, costly, and not widely available. An alternative is the measurement of urinary Cr clearance (CrCl). However, the required timed urine collection is cumbersome and prone to errors and the result needs time to be reported. Hence, estimation of GFR using methods that are practical and timely is desirable in all patients in general. This might be more important in critically ill patients as they have increased prevalence of kidney dysfunction [3] and frequently exhibit augmented renal clearance (ARC) [4, 5]. Hence, proper dosing of medications in these patients would enhance their therapeutic effect, reduce potential toxicities, and improve patient outcomes [6, 7].

Multiple equations have been produced to estimate GFR, including Cockroft–Gault (CG) [8], modification of diet in renal disease study (MDRD) [9], chronic kidney disease epidemiology collaboration (CKD-EPI) [10], and Jelliffe [11] equations. These equations are primarily based on serum Cr and various anthropometric data. They were mostly derived from patients who were not critically ill [8–10]. Hence, there are concerns regarding their use in the ICU setting [12]. Studies that tested the accuracy of these equations in estimating renal function in the ICU setting are not many. Some focused on certain patient groups, especially those with ARC [13, 14], while others had low number of patients [13, 15–17]. The objective of this study was to assess the performance of commonly used formulas that estimate GFR against measured urinary CrCl in critically ill patients with different degrees of kidney function.

## 2. Methods

### 2.1. Study Design

This is a substudy of the PermiT (Permissive Underfeeding versus Target Enteral Feeding in Adult Critically Ill Patients) trial (https://clinicaltrials.gov/ct2/show/ISRCTN68144998), a multicenter randomized controlled trial which compared permissive underfeeding (40–60% of caloric requirements) versus target feeding (70–100% of caloric requirements) in ICU patients with similar protein intake in both groups (November 2009 to September 2014) [18]. Eligible patients were those who received tube feeding within 48 hours of ICU admission, were expected to stay in the ICU >72 hours, and were not on high doses of vasopressors [18]. The trial found no difference in the primary outcome (90-day mortality: 27.2% vs. 28.9%, respectively; relative risk: 0.94, 95% CI, 0.76–1.16; *p*=0.58) [18]. The trial required serial 24-hour urine collection to measure nitrogen balance. In this retrospective study, we included the patients enrolled in the trial at King Abdulaziz Medical City-Riyadh who had at least one 24-hour urine collection for Cr, allowing CrCl (CrCl_24h-urine_) measurement. Patients with end-stage renal disease requiring dialysis and those with anuria for any other reasons were excluded. Subjects with missing variables needed for calculations of the different equations were also excluded. The original trial was approved by the Institutional Review Board of Ministry of National Guard Health Affairs, Riyadh, Saudi Arabia.

### 2.2. Data Collection

At baseline, we collected data on patients' demographics, chronic comorbid conditions, admission category (medical, surgical, and trauma), presence of traumatic brain injury, presence of sepsis on admission, Acute Physiology and Chronic Health Evaluation (APACHE II) score, Sequential Organ Failure Assessment (SOFA) score, use of mechanical ventilation, need for vasopressor therapy because of shock, daily caloric and protein intake, and laboratory results. We also obtained data about clinical outcomes, including mortality, duration of mechanical ventilation, and length of stay in the ICU and hospital.

In the study patients, urine was collected over 24 hours at baseline and then weekly as required by the trial when applicable. Measured CrCl_24h-urine_ was then calculated using the standard equation: (urine Cr × urinary flow in ml/min)/serum Cr, where urine and serum Cr were expressed in *µ*mol/L. To estimate GFR using different equations, we used the following variables taken on the same day of urine collection: age, weight, serum Cr, blood urea nitrogen, and albumin. In our laboratory, serum and urinary Cr concentrations were analyzed by a standardized Jaffe method (alkaline picrate reaction) traceable to isotopic dilution mass spectrometry using Abbott Architect c16000 platform.

### 2.3. Estimation of Kidney Function

We estimated GFR using CG [8], MDRD [9], CKD-EPI [10], and Jelliffe [11] equations. These different equations are described in Table 1. For CG equation, we entered the actual weight in one calculation (CG_actual-wt_), and if body mass index (BMI) ≥30 kg/m^2^, the ideal body weight (CG_ideal-wt_) and the adjusted body weight (CG_adjusted-wt_) were used in two calculations. We calculated the MDRD equation based on 4 (MDRD-4) and 6 variables (MDRD-6). Acute kidney injury in the enrollment day was assessed using the KDIGO classification [19].

### 2.4. Statistical Analysis

Continuous variables were reported as mean and standard deviation (SD). The coefficient of variation (SD/mean × 100) for CrCl_24h-urine_ and the estimated GFR were also calculated. Categorical data were presented as frequency with percentage. Chi square test was used to assess between-group differences in categorical variables. Student's *t* or ANOVA tests were used to assess between-group differences in continuous variables as indicated.

The performance of the GFR-estimating equations compared with CrCl_24h-urine_ was assessed in several ways. Correlations were reported using Spearman correlation coefficient (*r*). Bias represented the mean difference between CrCl_24h-urine_ and each of the equations estimating GFR [20]. Precision was defined as one SD of the bias [20]. Error was defined as double SD of the bias divided by the mean of the equation under study and CrCl_24h-urine_. An acceptable between-method error was defined as 30% or less [21]. Accuracy was defined as percentage of GFR estimations within ±15, ±30, and ±50% range of respective CrCl_24h-urine_ measurements. The 2002 Kidney Disease Outcomes Quality Initiative guidelines recommended that ≥90% of estimates be within 30% [22]. Bland–Altman plots were generated by plotting bias on the *Y*-axis and the mean of the equation under study and CrCl_24h-urine_ on the *X*-axis [23]. The limits of agreement (bias ± two SD of the bias) were shown in the plots.

The predictive performance of the different equations was assessed when CrCl_24h-urine_ was <30, 30–59.9, 60–130, and > 130 ml/min. We also assessed the ability (sensitivity) of the different equations to correctly classify CrCl_24h-urine_ within clinically relevant ranges (<30, 30–59.9, 60–130, and >130 ml/min). Moreover, Spearman correlation was calculated in selected subgroups of patients: age < versus ≥ 65 years, BMI < versus ≥ 30 kg/m^2^, APACHE II score < versus ≥ median value, which was 20, admission categories (medical, surgical, and nonoperative trauma), diagnosis of traumatic brain injury, presence of sepsis on ICU admission, baseline Cr < versus ≥ 110 *µ*mol, presence of AKI, and presence of ARC (baseline CrCl_24h-urine_ >130 ml/min/173 m^2^) [4, 5].

Tests were two-sided and statistical significance was determined at *p* < 0.05. Bias was considered significant if the null hypothesis (bias = 0) was rejected. Analyses were conducted using SAS version 9.2 (SAS Institute, Cary, NC) and SPSS version 15.

## 3. Results

### 3.1. Characteristics of Patients

Two hundred and thirty-seven patients were included in this study.  Table 2 describes their characteristics. The mean age was 45.0 ± 20.2 years, 74.7% were men, 32.5% were obese (BMI >30 kg/m^2^), 31.7% had diabetes, 98.7% required mechanical ventilation, and 26.2% had traumatic brain injury.

**Figure 1:**
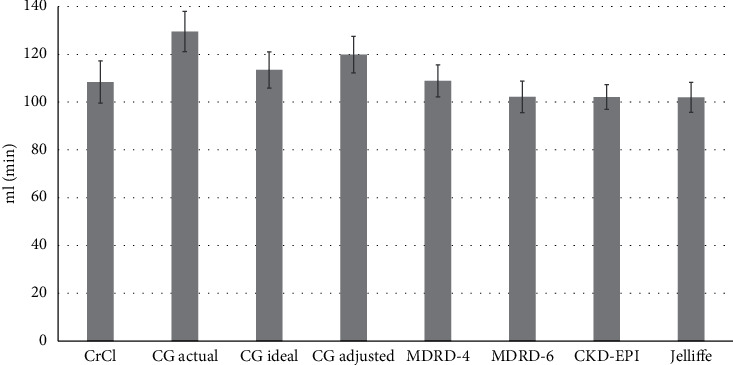
Mean values of the measured creatinine clearance by 24-hour urine collection (CrCl_24h-urine_) and estimated glomerular filtration rate by different equations. The first 237 24-hour urine samples were used in this analysis. Error bars represent 95% confidence interval. The difference between the different methods was significant (*p* < 0.001) by ANOVA test. CG: Cockroft–Gault equation; MDRD-4: 4-variable modification of diet in renal disease equation; MDRD-6: 6-variable modification of diet in renal disease equation, and CKD-EPI: chronic kidney disease epidemiology collaboration equation.

The baseline serum Cr was 100.8 ± 93.9 *µ*mol/L. Most patients (53%) had 24-hour urine collection once, 17.7% twice, 13.9% three times, and 15.2% four times. Thus, there were 453 measurements of urinary CrCl. Whereas 18.1% had AKI, 38.4% had ARC based on CrCl_24h-urine_ >130 ml/min at baseline. The measured CrCl_24h-urine_ and estimated GFRs based on the various equations are presented in Figure 1. The mean CrCl_24h-urine_ was 108.4 ± 68.9 ml/min in the first 237 measurements. The estimated GFR by the different equations were 129.6 ± 65.6 ml/min for CG _actual-wt_ (*p*=0.001), 113.5 ± 59.2 ml/min for CG_ideal-wt_ (*p*=0.39), 119.9 ± 59.9 for CG_adjusted-wt_ (*p*=0.053), 108.9 ± 52.5 ml/min for MDRD-4 (*p*=0.93), 102.2 ± 48.7 ml/min for MDRD-6 (*p*=0.27), 102.1 ± 40.4 ml/min for CKD-EPI (*p*=0.22), and 102.0 ± 49.3 ml/min for Jelliffe equation (*p*=0.24). However, precision was high for all equations.

### 3.2. Performance of the Equations Estimating GFR in the Whole Cohort

The performance of the different GFR-estimating equations against CrCl_24h-urine_ is described in Table 3. The correlations between the different equations and CrCl_24h-urine_ were significant (*p* < 0.001), with r ranging between 0.62 and 0.79. When using the first 237 urine measurements, bias was large and statistically significant for CG_actual-wt_ (21.1 ml/min), CG_adjusted-wt_ (11.5 ml/min), and MDRD-6 (-10.3 ml/min) equations. When using all 453 urine measurements, the bias was large and statistically significant for CG_actual-wt_ (27.4 ml/min), CG_ideal-wt_ (12.3 ml/min) CG_adjusted-wt_ (18.3 ml/min), MDRD-4 (7.1 ml/min), and MDRD-6 (−5.7 ml/min) equations. In both calculations, CKD-EPI and Jelliffe equations had no significant bias. The error was >70% for all equations.

The accuracy values for the different equations were generally modest. When using the first 237 urine measurements, accuracy within ±10% of CrCl_24h-urine_ ranged between 12.7% (CG_actual-wt_ equation) and 30.0% (CG_adjusted-wt_ equation). Accuracy within ±30% ranged between 47.4% (MDRD-6 equation) and 51.2% (CG_adjusted-wt_ equation). Accuracy within ±57.4% ranged between 12.7% (Jelliffe equation) and 75.1% (MDRD-6 equation). The accuracy values were similar when all 453 measurements were used in calculation (Table 3).

**Figure 2:**
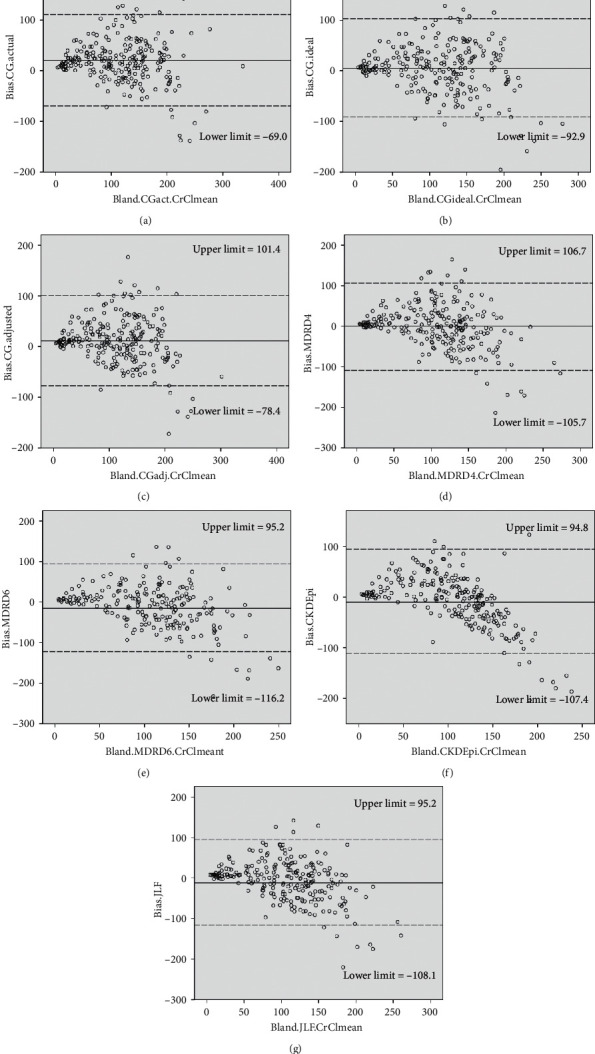
Bland–Altman plot of the creatinine clearance measured by 24-hour urine collection (CrCl_24h-urine_) versus the equations estimating glomerular filtration rate. (a) Cockroft–Gault equation using actual body weight; (b) Cockroft–Gault equation using ideal body weight; (c) Cockroft–Gault equation using adjusted body weight; (d) 4-variable modification of diet in renal disease equation; (e) 6-variable modification of diet in renal disease equation; (f) chronic kidney disease epidemiology collaboration equation; and (g) Jelliffe equation. The *X*-axis represents the difference between CrCl_24h-urine_ and the equation estimating glomerular filtration rate. The *Y*-axis represents the mean of CrCl_24h-urine_ and the equation estimating glomerular filtration rate. The solid line represents the bias (mean difference obtained across the range of values), whereas the dashed lines are the limits of agreement (±1.96 × standard deviation).

Bland–Altman plots are depicted in Figure 2. The limits of agreement were 111.3 and −69.0 ml/min for CG_actual-wt_, 103.0 and −92.9 for CG_ideal-wt_, 101.4 and −78.4 ml/min for CG_adjusted-wt_, 106.7 and −105.7 ml/min for MDRD-4, 95.2 and −116.2 ml/min for MDRD-6, 94.8 and −107.4 ml/min for CKD-EPI, and 95.2 and −108.1 ml/min for Jelliffe equations. Multiple points were outside the limits of agreement, which were wide for all equations.

### 3.3. Performance of the Equations Estimating GFR in Different Ranges of Urinary Creatinine Clearance and in Selected Subgroups of Patients

Correlation, bias, precision, and accuracy for the different equations are reported in Table 3 when CrCl_24h-urine_ < 30, 30–59.9, 60–130, and >130 ml/min using the 453 measurements, which were considered to be independent observations. Bias was significant for all equations except for CG_adjusted-wt_ equation when CrCl_24h-urine_ > 130 ml/min.

The sensitivity of GFR equations to correctly classify CrCl_24h-urine_ <30 ml/min was 44.7% for CG_actual-wt_, 71.1% for CG_ideal-wt_, 57.9% for CG_adjusted-wt_, 60.5% for MDRD-4, 64.5% for MDRD-6, 59.5% for CKD-EPI, and 60.5% for Jelliffe equation. The sensitivity to correctly classify CrCl_24h-urine_ 30–59.9 ml/min was 17.2% for CG_actual-wt_, 30.0% for CG_ideal-wt_, 31.0% for CG_adjusted-wt_, 31.0% for MDRD-4, 39.1% for MDRD-6, 13.8% for CKD-EPI, and 34.5% for Jelliffe equation. The sensitivity to correctly classify CrCl_24h-urine_ 60–129.9 ml/min was 59.5% for CG_actual-wt_, 60.8% for CG_ideal-wt_, 63.3% for CG_adjusted-wt_, 58.2% for MDRD-4, 59.7% for MDRD-6, 79.7% for CKD-EPI, and 63.3% for Jelliffe equation. The sensitivity to correctly classify CrCl_24h-urine_ ≥130 ml/min was 87.9% for CG_actual-wt_, 70.3% for CG_ideal-wt_, 79.1% for CG_adjusted-wt_, 60.4% for MDRD-4, 49.4% for MDRD-6, 45.1% for CKD-EPI, and 53.3% for Jelliffe equation.

Table 4 shows the Spearman correlations between the different GFR-estimating equations and CrCl_24h-urine_. The values of *r* were lowest in patients with the diagnosis of polytrauma, baseline Cr <110 *µ*mol and baseline CrCl_24h-urine_ >130 ml/min.

## 4. Discussion

In this study, we found that the commonly used equations to estimate GFR performed modestly against the measured urinary CrCl with high bias and accuracy within 30% present in approximately 50%. The equations with the highest sensitivity to correctly classify CrCl_24h-urine_ 30–59 and ≥130 ml/min, ranges where medication dose adjustment is frequently needed, were MDRD-6 and CG_actual-wt_.

Measuring GFR cannot be done routinely. Measured urinary CrCl is more widely available, but it may overestimate GFR because of Cr filtration and secretion; the latter can be affected by medications known to compete with active tubular secretion of Cr [24]. However, studies that compared CrCl_24h-urine_ with measured GFR in the ICU are limited. One study found that urinary CrCl with short collection times (1–2 h) had the highest correlation with measured GFR using inulin clearance (*r* = 0.921). The median bias for measured urinary CrCl was 11 mL/min/1.73 m^2^ for GFR <60ml/min, 24 mL/min/1.73 m^2^ for GFR 60–90ml/min, and 44 mL/min/1.73 m^2^ for GFR >90ml/min [17]. Another study evaluated 30 ICU patients with early AKI after complicated cardiac surgery and found low bias but high error when CrCl_24h-urine_ was compared with GFR measured by the infusion clearance of chromium-ethylenediaminetetraacetic acid [15]. The magnitude of this overestimation increased as GFR declined [15]. On the other hand, the commonly used equations to estimate GFR have their own shortcomings. They were mostly derived from outpatients with stable kidney function [8–10]. Only the Jelliffe equation was validated to assess GFR in a non-steady state as in critically ill patients [25]. Besides, studies that evaluated their use in the ICU settings had many limitations. Nevertheless, they generally found modest performance of GFR-estimating equations. A study of 360 critically ill patients who had stable serum Cr in one French hospital compared estimated GFR by equations that included CG, MDRD, and CKD-EPI, with CrCl_24h-urine._ The study found that the different equations tended to overestimate the CrCl for low eGFR values and to underestimate the CrCl for normal and high values [26]. In patients without ARC, the bias and precision were 11.3 and 25.3 ml/min for CKD-EPI, 18.8 and 31.7 ml/min for CG, and 22.5 and 34.6 ml/min, respectively [26]. Another study of 360 ICU subjects in Australia found that all tested equations (CG and CKD-EPI) showed limited agreement with 8-hour urinary CrCl [27]. CG_actual-wt_ corrected for body surface area had the lowest bias (-3.2 ml/min for indigenous and 8.2 ml/min for nonindigenous patients) [27]. However, CKD-EPI had the narrowest 95% confidence interval for limits of agreement in the Bland–Altman analysis [27]. A study of 111 patients without renal impairment in a Japanese ICU found that eGFR calculated using the Japanese equation correlated well with CrCl based on 8-hour urine collection (Spearman *r* = 0.75; *p* < 0.05) [28]. In contrast, the Bland–Altman plots showed that the bias of the two variables was −46.1 mL/min/1.73 m^2^, and the 95% limits of agreement were −128.9 to 36.7 mL/min/1.73 m^2^ [28]. In a study of 54 ICU patients with normal Cr, a statistically signiﬁcant, but poor, correlation was noted between CrCL by 8-hour urine collection and GFR estimated by CG, MDRD-4, and CKD-EPI (*r* = 0.20, 0.19, and 0.34, respectively) [16], The Bland–Altman plot showed poor agreement between pairs of comparisons (precision of 40.9, 39.8, and 33.4%, respectively) [16], When GFR-estimating equations were compared with measured GFR by inulin clearance in the ICU, CG, MDRD-6, MDRD-4, and CKD-EPI equations overestimated GFR (bias 24, 26, 37, and 13 mL/min/1.73 m^2^, respectively) [17]. However, CKD-EPI had the lowest bias likely due to its better performance when GFR >90 mL/min/1.73 m^2^ [17]. We evaluated seven different equations against CrCl_24h-urine_. All had significant bias, inadequate precision, high error, low accuracy, and wide agreement limits on the Bland-Altman plots. The correlations were moderate to strong nevertheless. Importantly, the sensitivity to correctly identify CrCl_24h-urine_ in the clinically important ranges (such as 30–59 and >130 ml/min) was low in general for all equations.

Studies on the performance of GFR-estimating equations in critically ill patients with AKI are scarce. One study evaluated 30 ICU patients with early AKI. GFR-estimating equations, CG, MDRD-4, and CKD-EPI equations, performed poorly when compared with measured GFR. The biases ranged from 7.4 ml/min for CG_actual-wt_ to 11.6 ml/min for MDRD-4 [15]. Additionally, the limits of agreement were wide for all the equations [15]. We found that the bias was generally high, but MDRD-6 had the lowest bias (28.4 ml/min). Jelliffe equation had the highest accuracy ±30%, but was only 35.3%. The correlations of studies equations with CrCl_24h-urine_ were fair. Moreover, MDRD-6 had the highest sensitivity (39.1%) to correctly classify CrCl_24h-urine_ 30–59.9 ml/min. This was mostly due to overestimation of GFR.

Other studies evaluated GFR-estimating equations in patents with ARC. A study of 390 patients with ARC in a surgical ICU in Belgium showed fair correlation between measured and estimated clearances (Spearman *r* = 0.34; *p* < 0.001 for CG equation and 0.29; *p* < 0.001 for MDRD-4 equation) [14]. The bias was −11.2 ml/min with limits of agreement (−131.7; 109.3 ml/min) for CG equation and −19.9 ml/min with limits of agreement (−170.4; 130.7 ml/min) for MDRD-4 [14]. Post hoc analysis of prospectively collected data in 86 patients with ARC at two tertiary ICUs in Australia and Portugal found that GFR estimated by CG, modified CG, MDRD-4, and MDRD-6 equations significantly underestimated CrCl_24h-urine_, with CG displaying the smallest bias [13]. The correlation was poor between CrCl_24h-urine_ and CG (*r* = 0.26, *p*=0.02) and MDRD-4 (*r* = 0.22, *p*=0.047), and neither had acceptable precision for clinical application in this setting [13]. CG estimates had the highest sensitivity for correctly identifying ARC (62%) [13]. In the current study, we found lower bias when CrCl_24h-urine_ ≥ 130 ml/min than lower ranges. CG_adjusted-wt_ had low bias (−0.7 ml/min), the highest accuracy± 30% (75.2%), and sensitivity to correctly classify CrCl_24h-urine_ ≥130 ml/min (79.1%). It should be noted that failure to correctly identify ARC may lead to subtherapeutic dosing of medications increasing the risk of treatment failure, emerging microbial resistance, prolonged ICU stay, and increased mortality [29].

GFR-estimating equations may not perform well in certain populations, such as the very elderly [30, 31], patients with diabetes [32], or those who have liver cirrhosis [33]. We studied subgroups of ICU patients and found that the correlation between CrCl_24h-urine_ and the different GFR-estimating equations was weak in patients with polytrauma, who commonly have ARC [34].

The findings of this study should be interpreted taking into consideration its strengths and limitations. The strength includes the prospective data collection, relatively large sample size, the study of seven GFR-estimating equations, and the evaluation of their performance using several methods. The limitations include being a single-center study and the use of CrCl_24h-urine_ instead of more accurate GFR measures (e.g., inulin, ^125^I-sodium iothalamate clearance or cystatin C-based equations). Serum cystatin C-based equations have been found to outperform serum creatine-based equations in estimating GFR in critically ill patients [35–37]. Moreover, CrCl_24h-urine_ is less accurate when kidney function is not steady and dysfunction is evolving [15], which is frequent in the ICU.

In conclusion, GFR-estimating equations that are commonly used in clinical practice had limited ability to properly estimate CrCl_24h-urine_ and likely true GFR. They had limited ability to correctly classify GFR into clinically relevant ranges that are usually needed to determine dosing of medications. The clinical significance of these findings needs to be studied further.

## Figures and Tables

**Table 1:** Renal function estimating equations.

*Cockcroft and Gault formula* (ml/min)
For males: [(140—age) × actual BW]/sCr × 72
For females: ([(140—age) × actual BW]/sCr × 72) × 0.85
sCr in mg/dL
The equation was calculated three times:
(1) Using actual BW for all patients
(2) Using actual BW for patients with BMI <30 kg/m^2^ and ideal BW for those with BMI >30 kg/m^2^
(3) Using actual BW for patients with BMI <30 kg/m^2^ and adjusted BW for those with BMI >30 kg/m^2^
*Ideal* BW
Males: 50 kg + 2.3 kg for each inch above 60 inches of height
Females: 45.5 kg + 2.3 kg for each inch above 60 inches of height
Adjusted BW = ideal BW + [0.4 × (actual BW—ideal BW)]

*Modification of diet in renal disease study equations* (ml/min/1.73 m^2^)
Four-variable equation: 175 × sCr ^−1.154^ × age^−0.203^ × 0.742 (if female)
Six-variable equation: 170 × sCr^−0.999^ × (Age)^−0.176^ × (0.762 if patient is female) × (BUN)^−0.170^× (albumin)^0.318^
sCr in mg/dL, albumin in g/dL, BUN in mg/dL; to convert BUN from mmol/L to mg/dL, divide by 0.3571

*Chronic kidney disease epidemiology collaboration (CKD-EPI) equations* (ml/min/1.73 m^2^)
For females with sCr ≤ 0.7: GFR = 144 × (sCr/0.7^)−0.329^ × (0.993)^age^
For females with sCr > 0.7: GFR = 144 × (sCcr/0.7)^−1.209^ × (0.993)^age^
For males with sCr ≤ 0.9: eGFR = 141 × (sCr/0.9)^−0.411^ × (0.993)^age^
For males with sCr > 0.9: eGFR = 144 × (sCr/0.9)^−1.209^ × (0.993)^age^
Age in years and sCr in mg/dL

*Jelliffe equation* (ml/min/1.73 m^2^)
For males: (98–16) × (age—20/20)/sCr
For females: [(98–16) × (age—20/20)/sCr] × 0.9
Age in years and sCr in mg/dL

BUN: blood urea nitrogen, BW: body weight, sCr: serum creatinine

**Table 2:** Characteristics and outcomes of the 237 patients in the study cohort.

	All patients *N* = 237
*Age*—(year), mean ± SD	45.0 ± 20.2
*Female sex*—no. (%)	60 (25.3)
*Height*—(cm), mean ± SD	166.3 ± 9.7
*Weight*—(kg), mean ± SD	78.2 ± 19.6
*Body mass index*—(kg/m^2^), mean ± SD	28.3 ± 7.2

*Chronic illnesses*—*no. (%)*	
Diabetes	75 (31.7)
Chronic respiratory disease	27 (11.3)
Chronic cardiac disease	23 (9.7)
Immunocompromised disorder	6 (2.5)
Chronic renal disease	6 (2.5)
Chronic liver disease	11 (4.6)

*Admission category, no. (%)*	
Medical	112 47.3)
Surgical	11 (4.6)
Nonoperative trauma	114 (48.1)
*Traumatic brain injury*—no. (%)	62 (26.2)
*Sepsis on admission*—no. (%)	51 (21.5)
*APACHE II*—mean ± SD	20.4 ± 8.1
*SOFA score day* 1—mean ± SD	10.0 ± 2.8
*Vasopressor use*—no. (%)	135 (57.0)
*Mechanical ventilation*—no. (%)	234 (98.7)

*Intervention group*—*no. (%)*	
Standard feeding	120 (50.6)
Permissive underfeeding	117 (49.4)
Total caloric intake (kcal/day)—mean ± SD	1143.6 ± 466.1
Total protein intake—(g/day) mean ± SD	55.9 ± 21.0

*Laboratory tests*	
Inclusion blood glucose—(mmol/L), mean ± SD	9.0 4.1
Creatinine—(*µ*mol/L), mean ± SD	100.8 ± 93.9
Bilirubin—(*µ*mol/L), mean ± SD	25.4 ± 39.2
Platelets—(10^9^/L), mean ± SD	214 ± 128
Albumin—(g/L), mean ± SD	28.8 ± 5.6

*Outcomes*	
Mechanical ventilation duration—(days), mean ± SD	13.0 ± 25.0
ICU LOS—(days), mean ± SD	15.9 ± 10.5
Hospital LOS—(days), mean ± SD	59.3 ± 83.1
90-day mortality	61 (25.7)
ICU mortality—no. (%)	38 (16.0)
Hospital mortality—no. (%)	56 (23.6)
ICU-acquired infections—no. (%)	96 (40.5)

SD: standard deviation; APACHE: acute physiology and chronic health evaluation; SOFA: sequential organ failure assessment; ICU: intensive care unit; LOS: length of stay.

**Table 3:** Predictive performance of equations estimating glomerular filtration rate compared with creatinine clearance (CrCl_24h-urine_) measured by 24-hour urine collection.

	CrCl_24h-urine_	CG_actual-wt_	CG_ideal-wt_	CG_adjusted-wt_	MDRD-4	MDRD-6	CKD-EPI	Jelliffe	
*Data from the first* 237 *urine collections (all* 237 *patients)*									
GFR estimate (mlH/min) ±SD	108.4 ± 68.9	129.6 ± 65.6	113.5 ± 59.2	119.9 ± 59.9	108.9 ± 52.5	102.2 ± 48.7	102.1 ± 40.4		102.0 ± 49.3
Coefficient of variation (%)	63.6	50.6	52.2	50.0	48.2	47.7	39.6		48.3
Correlation		0.77	0.71	0.75	0.63	0.62	0.67		0.66
Bias (ml/min)		21.1^*∗*^	5.0	11.5^*∗*^	0.5	−10.5^*∗*^	−6.3		−6.4
Precision (ml/min)		±46.0	±49.9	±45.9	±54.2	±53.9	±51.6		±51.9
Error (%)		77.3	90.0	80.4	99.7	100.4	98.0		98.6
*Accuracy*									
±15%		12.7	27.4	30.0	25.7	22.8	28.3	25.7	
±30%		49.4	48.1	51.2	48.9	47.4	48.9	49.4	
±50%		66.7	72.2	71.3	69.2	75.1	70.5	57.4	

*Data from the* 453 *urine collections (all* 237 *patients)*									
GFR estimate (ml/min) ±SD	102.7 ± 65.4	130.1 ± 65.9	114.9 ± 60.2	121.0 ± 60.6	109.8 ± 52.1	99.8 ± 47.1	103.2 ± 40.0	103.2 ± 49.3	
Coefficient of variation (%)	63.7	50.7	52.4	50.1	47.4	47.2	38.8	47.8	
Correlation		0.79	0.75	0.79	0.67	0.66	0.67	0.70	
Bias (ml/min)		27.4^*∗*^	12.3^*∗*^	18.3^*∗*^	7.1^*∗*^	−5.7^*∗*^	0.53	0.53	
Precision (ml/min)		±43.0	±44.3	±40.9	±49.1	±49.3	±47.2	±46.8	
Error (%)		73.9	81.5	73.2	92.5	96.1	91.8	90.9	

*Accuracy*									
±15%		24.1	27.8	29.1	26.7	27.5	28.0	26.7	
±30%		42.8	47.5	49.2	49.9	50.5	50.3	50.8	
±50%		60.0	69.3	66.2	69.3	73.5	69.8	72.2	

*Urine collections with CrCl*_24*h-urine*_ *<* 30 ml*/*min *(N* *=* 72)									
GFR estimate (ml/min) ±SD	13.3 ± 8.4	39.7 ± 25.7	30.8 ± 25.7	34.4 ± 25.6	34.4 ± 33.3	32.1 ± 28.4	38.2 ± 30.6	33.7 ± 27.9	
Coefficient of variation (%)	63.2	64.7	83.4	74.4	96.8	88.5	80.1	82.8	
Correlation		0.58	0.50	0.54	0.55	0.58	0.56	0.54	
Bias (ml/min)		26.4^*∗*^	17.6^*∗*^	21.1^*∗*^	21.1^*∗*^	18.7^*∗*^	25.0^*∗*^	20.4^*∗*^	
Precision (ml/min)		±21.9	±22.7	±21.8	±29.5	±24.4	±26.9	±24.4	

*Accuracy*									
±15%		1.4	16.7	5.6	12.5	13.1	11.1	8.3	
±30%		6.9	23.6	15.3	26.4	27.9	23.6	19.4	
±50%		11.1	36.1	23.6	36.1	39.3	30.6	31.9	

*Urine collections with CrCl*_24*h-urine*_ *30*–59.9 ml/min *(N* *=* *68)*									
GFR estimate (ml/min) ±SD	45.3 ± 8.6	85.6 ± 34.5	76.9 ± 37.3	80.4 ± 35.4	84.8 ± 40.6	73.8 ± 36.5	86.3 ± 29.5	76.0 ± 35.3	
Correlation		0.44	0.46	0.46	0.30	0.46	0.35	0.33	
Coefficient of variation (%)	19.0	40.3	48.5	44.0	47.9	49.5	34.2	46.4	
Bias (ml/min)		40.3^*∗*^	31.6^*∗*^	35.1^*∗*^	39.5^*∗*^	28.4^*∗*^	41.0^*∗*^	30.6^*∗*^	
Precision (ml/min)		±31.7	±34.3	±32.4	±38.9	±33.5	±27.6	±33.5	

*Accuracy*									
±15%		5.9	11.8	14.7	7.4	15.5	7.4	11.8	
±30%		16.2	26.5	25.0	20.6	32.8	14.7	35.3	
±50%		27.9	44.1	39.7	38.2	53.4	23.5	51.5	

*Urine collections with CrCl*_24*h-urine*_ 60–129.9 ml/min *(N* *=* *156)*									
	97.8 ± 20.5	134.2 ± 44.5	121.6 ± 43.8	126.6 ± 41.9	120.8 ± 37.6	107.7 ± 35.5	114.2 ± 26.4	111.7 ± 36.2	
Coefficient of variation (%)	21.0	33.2	36.0	33.1	31.1	33.0	23.1	32.4	
Correlation		0.37	0.38	0.40	0.12	0.18	0.32	0.23	
Bias (ml/min)		36.4^*∗*^	23.8^*∗*^	28.8^*∗*^	23.0^*∗*^	9.6^*∗*^	16.4^*∗*^	13.9^*∗*^	
Precision (ml/min)		±41.6	±40.6	±38.7	±40.7	±37.6	±20.7	±37.2	

*Accuracy*									
±15%		24.4	27.6	29.5	27.6	32.6	42.3	28.8	
±30%		44.2	46.2	49.4	53.8	54.5	61.5	55.8	
±50%		66.7	71.8	69.2	72.4	73.1	84.0	78.8	

*Urine collection with CrCl*_24*h-urine*_ *>* 130 ml/min *(N* *=* *157)*									
GFR estimate (ml/min) ±SD	173.3 ± 41.6	186.6 ± 44.2	163.3 ± 37.6	172.6 ± 34.3	144.1 ± 32.3	130.3 ± 30.5	129.4 ± 16.3	138.4 ± 31.3	
Coefficient of variation (%)	24.0	23.7	23.0	19.9	22.4	23.4	12.6	22.6	
Correlation		0.29	0.20	0.28	0.22	0.14	0.14	0.20	
Bias (ml/min)		13.3^*∗*^	−10.0^*∗*^	−0.7	−29.2^*∗*^	−43.7^*∗*^	−44.0^*∗*^	−34.9^*∗*^	
Precision (ml/min)		±51.3	±50.0	±45.8	±46.7	±48.2	±42.5	±46.8	

*Accuracy*									
±15%		42.0	40.1	45.9	40.8	33.1	30.6	39.5	
±30%		69.4	68.8	75.2	69.4	57.4	66.9	66.9	
±50%		89.8	93.0	94.3	94.9	88.5	93.6	93.0	

CKD-EPI: chronic kidney disease epidemiology collaboration; CG: Cockroft-Gault; CrCl_24h-urine_: creatinine clearance measured by 24-hour urine collection; GFR: glomerular filtration rate; MDRD: modification of diet in renal disease study; SD: standard deviation. CG_actual-wt_: the CG equation was calculated using actual body weight. CG_ideal-wt_: the CG equation was calculated using ideal body weight. CG_adjusted-wt_: the CG equation was calculated using adjusted body weight. MDRD-4: the MDRD equation was calculated using four variables. MDRD-6: the MDRD equation was calculated using six variables. ^*∗*^*p* < 0.05 using one-sided *t* test indicating that the bias was significant.

**Table 4:** Correlation between equations estimating glomerular filtration rate and creatinine clearance measured by 24-hour urine collection.

	Spearman correlation (*r*)						
	CG_actual-wt_	CG_ideal-wt_	CG_adjusted-wt_	MDRD-4	MDRD-6	CKD-EPI	Jelliffe
Age ≥ 65 years (*N* = 53)	0.70	0.62	0.67	0.60	0.61	0.67	0.61
Age < 65 years (*N* = 184)	0.71	0.62	0.69	0.57	0.56	0.57	0.58
BMI ≥ 30 kg/m^2^ (*N* = 77)	0.82	0.82	0.83	0.76	0.73	0.81	0.78
BMI < 30 kg/m^2^ (*N* = 160)	0.73	0.73	0.73	0.61	0.64	0.63	0.64
Admission category: medical (*N* = 112)	0.79	0.72	0.78	0.63	0.66	0.70	0.67
Admission category: surgical (*N* = 11)	0.74	0.58	0.68	0.70	0.48	0.66	0.67
Admission category: nonoperative trauma (*N* = 114)	0.54	0.37	0.46	0.34	0.30	0.22	0.32
APACHE II ≥ 20 (*N* = 118)	0.77	0.74	0.77	0.68	0.67	0.71	0.71
APACHE II < 20 (*N* = 119)	0.71	0.59	0.67	0.48	0.46	0.53	0.55
Diabetes (*N* = 75)	0.78	0.70	0.77	0.65	0.62	0.67	0.68
No diabetes (*N* = 162)	0.70	0.62	0.68	0.52	0.52	0.57	0.55
Sepsis admission (*N* = 51)	0.76	0.74	0.77	0.62	0.64	0.69	0.65
No sepsis on admission (*N* = 186)	0.74	0.66	0.72	0.59	0.57	0.61	0.62
Traumatic brain injury (*N* = 62)	0.56	0.39	0.49	0.41	0.43	0.21	0.32
No traumatic brain injury (*N* = 175)	0.78	0.72	0.77	0.64	0.63	0.70	0.67
SOFA renal > 0 (Cr ≥ 110 *μ*mol/L) (*N* = 49)	0.90	0.86	0.90	0.82	0.82	0.81	0.85
SOFA renal = 0 (Cr < 110 *μ*mol/L) (N = 188)	0.64	0.54	0.62	0.41	0.43	0.45	0.46
Acute kidney injury on admission (*N* = 43)	0.79	0.68	0.75	0.68	0.63	0.68	0.70
No acute kidney injury on admission (*N* = 188)	0.65	0.56	0.63	0.41	0.45	0.46	0.47
Baseline CrCl_24h-urine_ ≥ 130 ml/min (*N* = 91)	0.38	0.26	0.36	0.27	0.17	0.22	0.23
Baseline CrCl_24h-urine_< 130 ml/min (*N* = 146)	0.70	0.68	0.70	0.62	0.62	0.71	0.64

APACHE: acute physiology and chronic health evaluation; BMI: body mass index; CKD-EPI: chronic kidney disease epidemiology collaboration; CG: Cockroft-Gault; CrCl_24h-urine_: creatinine clearance measured by 24-hour urine collection; GFR: glomerular filtration rate; MDRD: modification of diet in renal disease study. CG_actual-wt_: the CG equation was calculated using actual body weight. CG_ideal-wt_: the CG equation was calculated using ideal body weight. CG_adjusted-wt_: the CG equation was calculated using adjusted body weight. MDRD-4: the MDRD equation was calculated using four variables. MDRD-6: the MDRD equation was calculated using six variables.

## Data Availability

The datasets used and/or analyzed during the current study are available from the corresponding author on reasonable request.
